# Body Stalk Anomalies in Pigs: Current Trends and Future Directions in Classification

**DOI:** 10.3390/ani15030460

**Published:** 2025-02-06

**Authors:** Nieves Martín-Alguacil, José Miguel Cozar, Luis J. Avedillo

**Affiliations:** Departmental Section of Anatomy and Embryology, School of Veterinary Medicine, Universidad Complutense de Madrid, 28040 Madrid, Spain; jcozar@ucm.es (J.M.C.); luiavedi@ucm.es (L.J.A.)

**Keywords:** BSA classification, LBWC, SPLBWC, SPBWC, STBWC, SSBWC, animal model, pig

## Abstract

Body stalk anomaly (BSA) is a complex congenital condition characterized by defects in the body wall, skeletal abnormalities, and an absent or abnormal umbilical cord (UC). A classification system for BSA, based on wall and skeletal abnormalities, is proposed and includes eight types observed in porcine models. These classifications consider combinations of thoracoabdominoschisis, abdominoschisis, anal atresia, genitourinary anomalies, and skeletal defects, including extreme retroflexion, scoliosis, vertebral agenesis, hemivertebrae, and thoracic or limb anomalies. While previous studies often treat limb-body wall complex (LBWC) as a separate condition, this study includes cases with co-occurring limb and spinal defects in a new classification, spinal-limb-body wall complex (SPLBWC). Additional skeletal classifications—spine-body wall complex (SPBWC), sternal-body wall complex (STBWC), and sternal-spinal-body wall complex (SSBWC)—are introduced to account for variations in structural anomalies. Nonstructural skeletal anomalies such as deformities, amputations, and arthrogryposis are excluded from the structural classifications. This comprehensive system, based on existing human and porcine models, provides a framework for the systematic categorization of BSA variations. Such an approach supports accurate diagnosis, enhances understanding of developmental defects, and improves clinical management and research outcomes in both veterinary and human medicine.

## Simple Summary

Body stalk anomaly (BSA) is a rare birth defect that affects the body wall, skeleton, and umbilical cord. To better understand and describe these anomalies, researchers studied cases in pigs and created a new classification system. This system divides BSA into eight types based on the specific combinations of defects, such as body wall openings, umbilical cord problems, and skeletal deformities, such as curved spines or missing bones. For example, some types include problems with both the spine and limbs, while others may affect the sternum or ribs. A related condition, limb-body wall complex (LBWC), is often treated as a separate category, but this study found that all piglets with limb defects also had spinal problems, so they were grouped into a new classification called the spinal-limb-body wall complex (SPLBWC). Other classifications were created for defects affecting the spine, sternum or both. This system helps doctors and researchers describe these abnormalities more accurately, making them easier to diagnose, study, and, where possible, treat. By improving our understanding of these conditions in animals, we can also learn more about similar defects in humans and find better ways to help those affected.

## 1. Introduction

Body stalk anomalies (BSA) are considered when there is a body wall anomaly, skeletal abnormalities, and the umbilical cord are anomalous, absent, or rudimentary [1], and limb-body wall complex (LBWC) is when there are body wall and structural limb anomalies with or without craniofacial abnormalities [1,2,3,4,5]. These anomalies are thought to result from early embryonic developmental abnormalities, in particular, abnormal embryonic folding and mesodermal tissue fusion [6]. In humans, BSA is often diagnosed prenatally through ultrasound and is associated with a high rate of mortality due to the complexity of the anomalies and the challenges in providing effective treatment [7,8,9,10]. While BSA has been extensively studied in humans, there is limited research on its occurrence and presentation in animal models [6,11,12]. Pigs are valuable models for studying human congenital anomalies due to their physiological and anatomical similarities to humans [13]. However, research on BSA in pigs is limited, with classification systems often lacking the consistency and depth necessary to capture the variability observed in these anomalies. This limitation stems primarily from the relatively low number of documented cases in veterinary research compared to the extensive databases available in human medicine. In human healthcare, congenital anomalies like BSA are rigorously studied due to their direct clinical and surgical implications, leading to more comprehensive classification frameworks. By contrast, the limited focus on congenital anomalies in pigs, primarily viewed through an agricultural or production lens, results in an underrepresentation and a narrower understanding of these anomalies. Moreover, the environmental conditions under which pigs typically give birth add another layer of complexity. Farrowing in farm settings is often less controlled than in research and medical facilities, with limited opportunities for close observation or immediate postnatal examination. This can result in congenital malformations like BSA being overlooked, especially if piglets do not survive long after birth. Such conditions also make it challenging to distinguish congenital anomalies from injuries incurred during or after delivery, such as crushing, trampling, or other trauma caused inadvertently by the sow. This confusion can lead to misdiagnosis, where true congenital anomalies are attributed to physical harm, further skewing the available data. Additionally, the nature of farming practices, where individual monitoring of each piglet is not always feasible, contributes to a lack of systematic reporting. Training farm personnel to recognize specific signs of BSA and other malformations and establishing protocols for detailed documentation are crucial steps toward bridging this gap.

The first classification of body stalk anomalies (BSA) in animals was conducted using pigs by Martín-Alguacil and Avedillo [6]. The proposed classification system differentiates between the types of body wall anomalies (abdominoschisis and thoracoabdominoschisis) and limb anomalies. According to Rittler et al. [14], limb structural anomalies are defined based on embryological failures. Nonstructural anomalies are considered to result from the actions of amniotic bands (AB) and/or fetal movement restrictions (FMR), including conditions such as arthrogryposis. Structural anomalies include amelia and/or phocomelia of the pelvic limbs, while nonstructural anomalies encompass thoracic limb phocomelia, arthrogryposis, ankyloses, and/or anomalous rotation [6]. The classification identifies four types of BSA presentations: BSA Type I: Piglets with spinal and umbilical cord (UC) anomalies, thoracoabdominoschisis, anal atresia, and/or other internal organ structural anomalies, along with structural limb anomalies. BSA Type II: Piglets with spinal and UC anomalies, thoracoabdominoschisis, anal atresia, and/or other internal organ structural anomalies with nonstructural limb anomalies. BSA Type III: Piglets with spinal and UC anomalies, abdominoschisis, anal atresia, and/or other internal organ structural anomalies and structural limb anomalies. And BSA Type IV: Piglets with spinal and UC anomalies, abdominoschisis, anal atresia, and/or other internal organ structural anomalies, with nonstructural limb anomalies. Additionally, two types of limb-body wall complex (LBWC) are distinguished: Type I, characterized by thoracoabdominoschisis and structural limb anomalies, corresponding to BSA Type I, and Type II, characterized by abdominoschisis and structural limb anomalies, corresponding to BSA Type III [6]. This study marked a significant milestone, as it provided a structured framework for studying BSA in an animal model, facilitating comparisons across cases and setting the stage for subsequent research. Building upon this foundational work, Martín-Alguacil later expanded the classification system to encompass eight types of BSA in human medicine. This expanded system reflects the broader spectrum of anomalies observed in clinical practice and underscores the complexity of these malformations in humans. By leveraging insights gained from animal models like pigs, Martín-Alguacil [2] demonstrated the translational value of comparative research in understanding and categorizing congenital anomalies. For the classification of human BSA cases, four more distinct types are defined: BSA Type V includes the presence of thoracoabdominoschisis, spinal anomaly, anomalous UC, and structural limb anomaly; BSA Type VI includes thoracoabdominoschisis, spinal anomaly, anomalous UC, and nonstructural limb anomaly; BSA Type VII includes abdominoschisis, spinal anomaly, anomalous UC, and structural limb anomaly; and BSA Type VIII includes abdominoschisis, spinal anomaly, anomalous UC, and nonstructural limb anomalies or no limb anomaly [2].

The primary objective of this study is to present and analyze thirty cases of BSA in pigs with varying degrees of severity. By comparing these cases with the existing classification system for human BSA, we aim to refine and expand the current understanding of the condition. This comparative analysis will highlight the similarities and differences in the presentation and progression of BSA between pigs and humans, offering a broader perspective on the condition. The study will enhance the limited body of knowledge on BSA in animals, particularly in pigs [15,16]. By comparing porcine and human cases, we can identify commonalities and differences that may inform both veterinary and human medical practices. Implementing a classification and grading system for BSA can lead to more accurate diagnoses, personalized treatments, better prognostic predictions, and enhanced research opportunities, benefiting both human and veterinary medicine.

## 2. Materials and Methods

All the malformations included in this study were identified sporadically between 2002 and 2024. The animals were obtained from a pig farming association based in Toledo, Spain, comprising 15 pig production units with an average annual census of 6500 breeding sows operating in a closed production cycle. Thirty piglets with anomalous spinal curvature and significant abdominal organ evisceration were selected for analysis. Twenty of these animals had been studied previously [6,15,16,17]. All animals were obtained in accordance with European Union regulations (Directive 2010/63/EEC) and Spanish legislation (RD 53/2013). The study was carried out at the Laboratory for the Study of Congenital Malformations, located in the Departmental Section of Anatomy and Embryology, School of Veterinary Medicine, Universidad Complutense de Madrid, Madrid, Spain. Comprehensive gross evaluations of the thirty specimens were carried out using conventional anatomical methods. This study also included radiographic (X-ray) examinations, computerized axial tomography scans, and 3D reconstructions to further characterize the anomalies. This study categorized body wall anomalies into abdominoschisis and thoracoabdominoschisis and provided a detailed characterization of the anomalies affecting the limbs, spine, sternum, and internal organs. Structural limb anomalies included amelia and pelvic limb phocomelia, while nonstructural limb anomalies included thoracic limb phocomelia, arthrogryposis, ankylosis, and/or limb anomalous rotation. Structural spinal anomalies were defined as extreme retroflexion of the spine, extreme scoliosis, hemivertebrae, vertebral agenesis, and other structural tail anomalies. In addition, structural sternal anomalies included total or partial sternal splitting, sternal hypoplasia, and sternal agenesis. A fetus was classified as having a body stalk anomaly (BSA) if it had the following clinical features: a body wall anomaly, skeletal anomalies, and an abnormal, absent, or rudimentary umbilical cord. In addition, a fetus was classified as having a limb-body wall complex (LBWC) if the body wall anomalies were associated with structural limb anomalies; as having spinal-body wall complex (SPBWC) if the body wall anomalies were associated with structural spinal anomalies; as having spinal-limb and body wall complex (SPLBWC) if the body wall anomalies were associated with structural spinal and limb anomalies; as having sternal-body wall complex (STBWC) if the body wall anomalies were associated with structural sternum anomalies; and as having sternal-spinal-body wall complex (SSBWC).

## 3. Results

**Figure 1:**
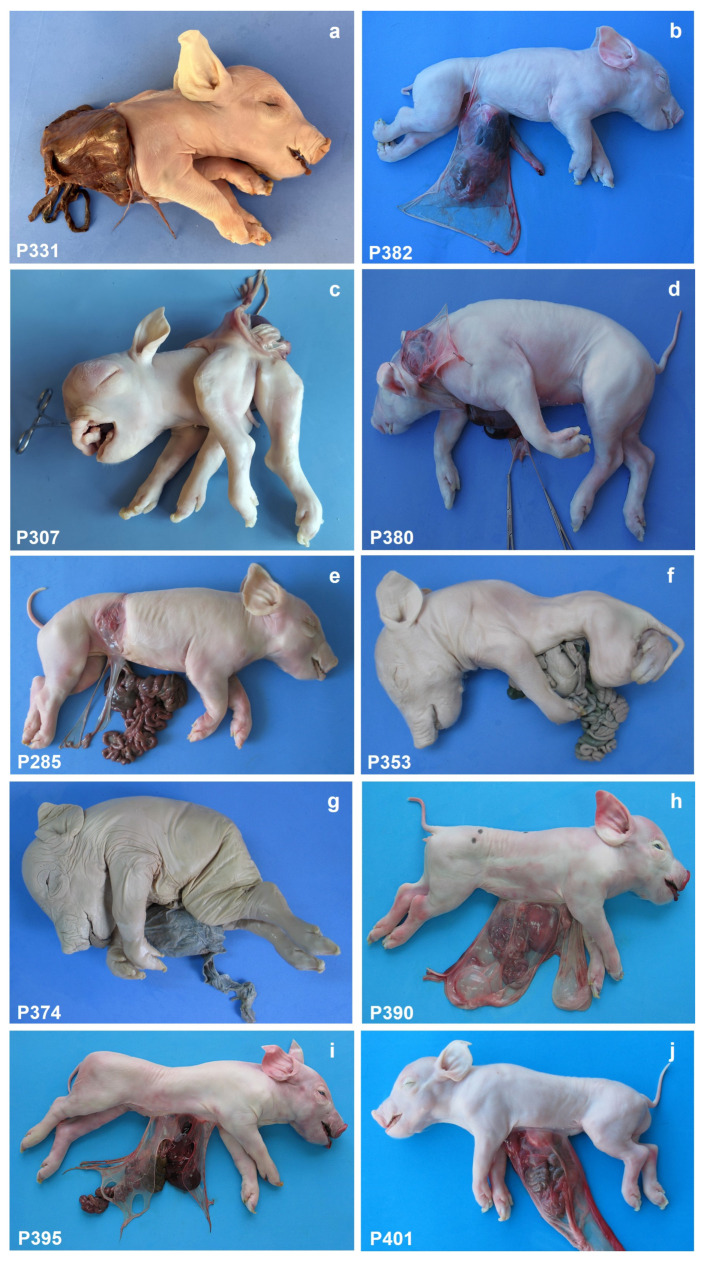
(**a**) Lateral view of piglet 331, showing thoracoabdominoschisis. Thoracic and abdominal organs are exposed. Extreme retroflexion of the spine. The lungs are hypoplastic with normal lobulation. Collapsed esophagus and trachea. Scattered umbilical vessels. Arthrogryposis and ankylosis of the pelvic limbs. Anal atresia. Unilateral left renal agenesis and hypoplastic genitals. There are no amniotic adhesions. (**b**) Lateral view of piglet 382, showing right lateral abdominoschisis. Exteriorization of the liver, stomach, duodenum, jejunum, cecum, and part of the colon, spleen, cranial third of the urinary bladder, and right testicle. Kyphosis and angulated tail. Sparse and short umbilical cord with 3 vessels. Arthrogryposis in hind limbs. Right unilateral cryptorchidism. Presence of amniotic adhesion on the right side, adjacent to the body wall defect. (**c**) Lateral view of piglet 307, showing thoracoabdominoschisis. Extreme retroflexion of the spine. The lungs are hypoplastic, with normal lobulation. Collapsed esophagus and trachea. Scattered umbilical vessels. Arthrogryposis and ankyloses in pelvic limbs. Complete palatoschisis and bilateral cheiloschisis; hydroanacephaly; ocular structure is lost in both orbits. Ocular malformations: Degeneration of both eyeballs. Eyelid edema/bilateral subpalpebral edema. (**d**) Lateral view of piglet 380, showing thoracoabdominoschisis. Externalization of part of the liver and jejunal loops. Spina bifida occulta C1–C5, hypoplasia of the vertebral arches T4–T6, and agenesis of the vertebral arches T7–T15. Sternoschisis and sternal hypoplasia (presence of 3 sternites on the right side and 4 sternites on the left side). Hypoplasia of the right ribs C7–C12 and left ribs C6–C10. Presence of right lateral diaphragmatic hernia. Short umbilical cord with 3 vessels. Arthrogryposis in the left forelimb. No atresia or anal stenosis. Left caudolateral cranioschisis with exencephaly. Hypoplasia of the left auricle. Left blepharitis. Left exophthalmia. Presence of amniotic adhesion in the cranial dysraphic defect. (**e**) Lateral view of piglet 285, showing abdominoschisis. Moderate scoliosis due to the presence of a hemi-vertebra (T14). Right, 14 ribs rotated and hypoplasic, and left, 15 ribs were bifid. Presence of abdominal wall dysplasia at the level of the right flank, dorsal to the body wall closure defect, is apparently not related to it. There are amniotic adhesions related to this area. Scattered umbilical vessels. Arthrogryposis of the right hind limb. (**f**) Lateral view of piglet 353, showing abdominoschisis. Presence of kypholordoescoliosis and angled tail. Scattered umbilical vessels. Arthrogryposis in all four limbs. The hindlimbs were hypoplastic and arthrogrypotic but otherwise completely developed and were held crosswise under the pelvis in an unusual, “Buddha-like” sitting position, characterized by external rotation with flexion-abduction of the hip joints, extreme flexion of the knee joints, extension of the hock joints, and flexion of the digits. Presence of secondary palatoschisis and mandibular brachygnathia. (**g**) Lateral view of piglet 374, showing abdominoschisis. Presence of kyphosis and sacrococcygeal agenesis. Scattered umbilical vessels. Arthrogryposis in all four limbs. (**h**) Lateral view of piglet 390, showing thoracoabdominoschisis. Externalization of the heart, liver, part of the stomach, duodenum, and jejunal loops. Sternoschisis and sternal hypoplasia. Scattered umbilical cord with 3 vessels. (**i**) Lateral view of piglet 395, showing thoracoabdominoschisis. Exteriorization of the liver, stomach, duodenum, jejunum, ileum, cecum, and part of the colon. Bifid sternum (V-shaped). Scattered umbilical cord with 3 vessels. (**j**) Lateral view of piglet 401, showing thoracoabdominoschisis. Externalization of the heart, liver, part of the stomach, duodenum, and jejunal loops.

**Figure 2:**
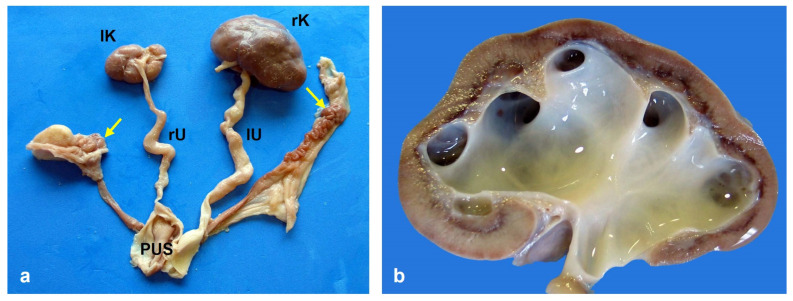
(**a**) Anatomical preparation of the urogenital system of piglet P79. There is a persistent urogenital sinus (PUS) into which the reproductive tract and both ureters (right, rU, and left, lU) are very dilated (hydroureters). The right kidney (rK) presents hydronephrosis, and the left kidney (lK) is hypoplastic. There is no urinary bladder. The ovaries (arrows) are hypoplastic, as is the entire reproductive tract. (**b**) Sagittal view of the right kidney. Note the presence of hydronephrosis and the reduced thickness of the renal cortex.

**Figure 3:**
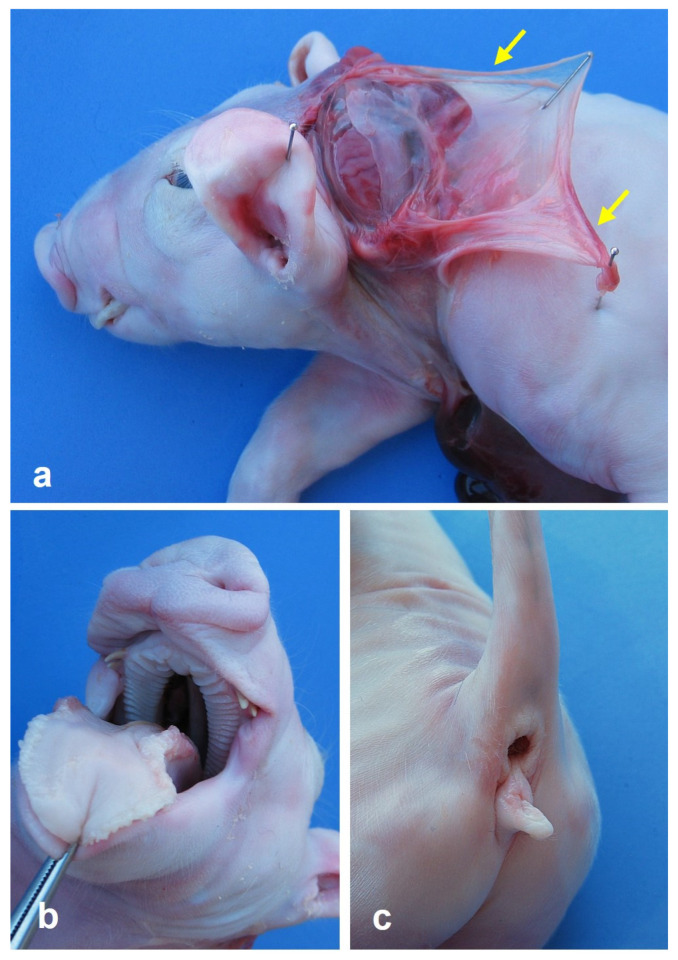
Case P380. (**a**) Left caudolateral view of piglet head, showing caudolateral cranioschisis with exencephaly, left blepharitis and left exophthalmia and hypoplasia of the left auricle. Note the presence of amniotic adhesion in the cranial dysraphic defect (arrows). (**b**) View of the roof of the oral cavity of piglet head; note the presence of palatoschisis. (**c**) Left caudolateral view of the perineal region.

Thirty stillborn crossbred (Landrace-Large White-Pietrain) piglets and the only abnormal members of their litter, 23 females and 7 males, were selected for the study. A summary of all the abnormalities observed in the 30 pigs is given in Table 1. The ten new cases are shown in Figure 1. After careful examination, anal atresia was not observed in 10 piglets. All these 10 pigs had some type of urogenital abnormality, which was also present in P120, P184, and P382, although the anus was normal in these three animals. Figure 2 shows the urogenital abnormalities observed in case P79: persistent urogenital sinus, hydroureters and hydronephrosis of the right kidney, hypoplastic left kidney, absent urinary bladder, and the uterus connected to the urogenital sinus, and the genitals were hypoplastic. Craniofacial anomalies were present in five cases: P160 had complete palatoschisis, P307 had palatoschisis and bilateral cheiloschisis, and P353 and P380 had cleft palate (secondary palatoschisis). In addition, case P380 presented left caudolateral cranioschisis with exencephaly, left blepharitis, and exophthalmia (Figure 3).

**Figure 4:**
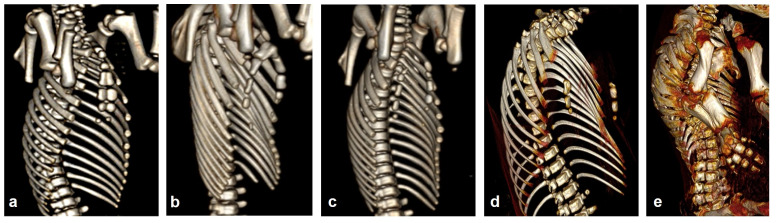
Right ventrolateral view of thoracic cavity skeleton on 3D-VR CT reconstructions showing the sternums of five of the piglets studied. (**a**) Piglet 382 with a normal sternum was used as a control. (**b**) Piglet 395, bifid sternum. (**c**) Piglet 390, complete sternoschisis. (**d**) Piglet 380, complete sternoschisis with agenesis of caudal sternites. (**e**) Piglet 353, sternal hypoplasia.

**Figure 5:**
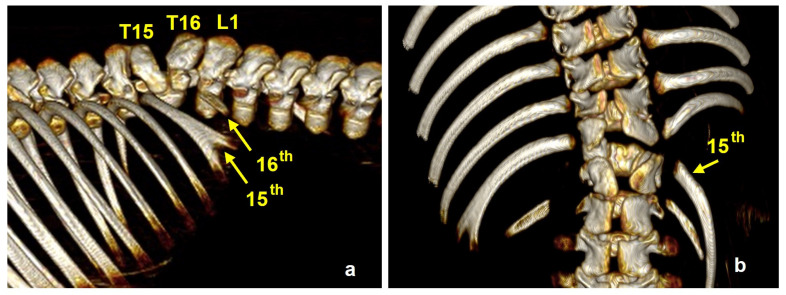
3D-VR CT reconstructions of piglet P285. (**a**) Right lateral and (**b**) dorsal view of the spine. Note the right 14th rib was rotated and hypoplasic and the left 15th rib was bifid.

Thoracoabdominoschisis was observed in eighteen animals (P17, P79, P168, P208, P226, P227, P228, P240, P264, P267, P278, P283, P307, P331, P380, P390, P395, and P401) with evisceration of all thoracic and abdominal organs. Eight piglets had abdominoschisis (P63, P65, P76, P120, P134, P285, P353, and P374), and four piglets (P160, P184, P241, and P382) had right lateral abdominoschisis with evisceration of all abdominal organs and extreme scoliosis. The abdominal organs were completely exposed in a large extraembryonic coelomic cavity covered only by the chorion. The sternum was cleft in nine piglets (P226, P227, P228, P267, P283, P380, P390, P395, and P401). The different sternal defects are shown in Figure 4. In P285, the right fourteenth rib was rotated and hypoplasic, the left fifteenth rib was bifid (Figure 5), and in P353, the sternum was hypoplasic (Figure 4). 

**Figure 6:**
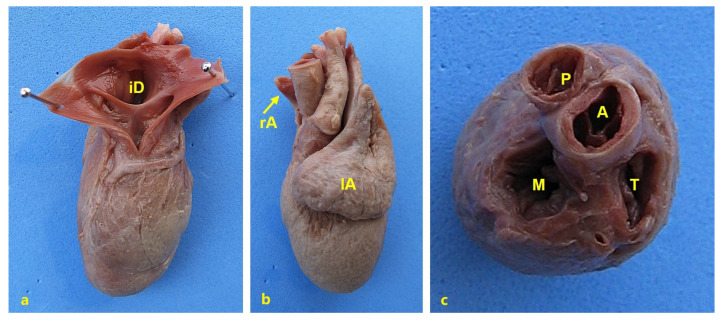
(**a**) Caudal view of the heart of piglet 401, after sectioning of the venae cavae. Note the large interatrial defect (iD). (**b**) Cranial view of the same heart showing the large left atrial (lA) hyperplasia and right atrial (rA) hypoplasia. (**c**) Dorsal view of the cardiac base of the same heart; note the tricuspid valve (T) stenosis. A, aorta; M, mitral valve; P, pulmonary trunk.

**Figure 7:**
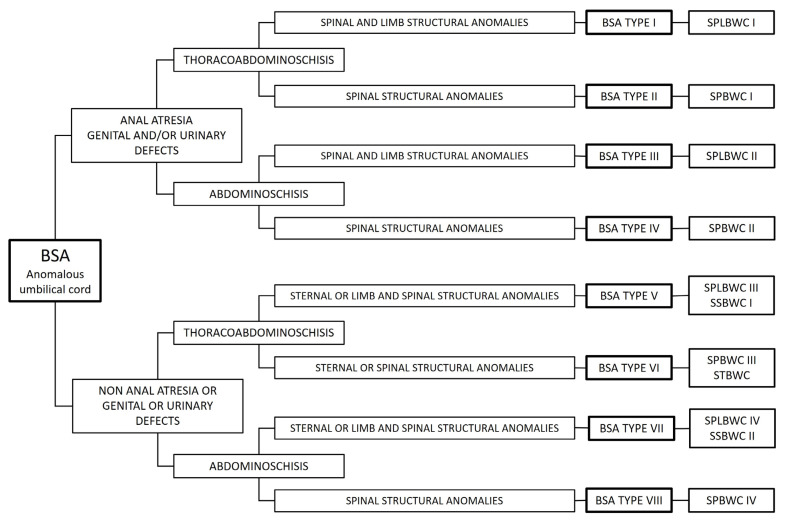
Body stalk anomalies classification: BSA, body stalk anomalies; SPBWC, spinal-body wall complex; SPLBWC, spinal-limb and body wall complex; SSBWC, sternal-spinal-body wall complex; STBWC, sternal-body wall complex.

Cardiac anomalies were observed in cases P226, P227, P228, P264, P267, P283, P390, and P401. The cardiac anomalies present in P401 are shown in Figure 6. Extreme retroflexion of the spine was observed in eight animals (P17, P79, P168, P208, P240, P278, P307, and P331), extreme scoliosis in two cases, and other structural anomalies of the spine or tail were observed in an additional nine animals (Table 1). Hemivertebrae or partially segmented hemivertebrae were observed in cases of extreme spinal flexion. Structural limb anomalies were observed in six cases (P17, P65, P79, P160, P240, and P278) and nonstructural limb anomalies in 13 animals (P76, P134, P168, P184, P208, P241, P285, P307, P331, P374, P380, and P382), and 11 animals had normal limbs. The umbilical vessels were found to be dispersed in most of the piglets examined. A single umbilical artery (SUA) was identified in P65, P208, and P240. The left umbilical artery was hypoplastic in all other cases. The amniotic membrane was attached to the skin margin of the thoracoabdominal or abdominal fissure. AB was observed in six piglets (P65, P76, P134, P285, P380, and P382). After detailed anatomical examination, the piglets were classified according to the Martín Alguacil [2] classification for human BSA cases but considering for the study three types of structural skeletal anomalies: spinal, sternal, and limb structural anomalies. The cases were classified as follows and presented in Figure 7: BSA Type I: Piglets with thoracoabdominoschisis, anomalous UC, anal atresia, urinary and/or genital anomaly, and limb and spinal structural skeletal anomalies. BSA Type II: Piglets with thoracoabdominoschisis, anomalous UC, anal atresia, urinary and/or genital anomalies, and structural spinal anomalies. BSA Type III: Piglets with abdominoschisis, anomalous UC, anal atresia, urinary and/or genital anomalies, and limb and spinal structural anomalies. BSA Type IV: Piglets with abdominoschisis, anomalous UC, anal atresia, urinary and/or genital anomalies, and spinal structural anomalies. BSA Type V: Piglets with thoracoabdominoschisis, anomalous UC, and sternal and spinal structural anomalies. BSA Type VI: Piglets with thoracoabdominoschisis, anomalous UC, and sternal or spinal structural anomalies. BSA Type VII: Piglets with abdominoschisis, anomalous UC, and sternal and spinal structural anomalies. BSA Type VIII: Piglets with abdominoschisis, anomalous UC, and spinal structural anomalies. Two cases (P63 and P120) were diagnosed as omphalocele and were not classified as BSA.

In the BSA Type I group, piglets with spinal and limb structural abnormalities were classified as spinal-limb and body wall complex I (SPLBWC I). In the BSA Type II group, piglets with spinal structural anomalies and no other skeletal anomalies were classified as spinal-body wall complex I (SPBWC I). In the BSA Type III group, piglets with structural spinal and limb anomalies were classified as spinal-limb and body wall complex II (SPLBWC II). In the BSA Type IV group, animals with spinal structural anomalies and no other skeletal anomaly were classified as spinal-body wall complex II (SPBWC II). There were no animals with structural abnormalities of the limbs as the only structural skeletal defect. In the BSA Type V group, piglets with structural sternal and spinal anomalies were classified as sternal-spinal-body wall complex III (SSBWC I). In the BSA Type VI group, animals with structural spinal anomalies and no other skeletal anomaly were classified as spinal-body wall complex III (SPBWC III), and animals with structural sternal defects and no other skeletal anomaly were classified as sternal-body wall complex (STBWC). In the BSA Type VII group, animals with structural sternal and spinal anomalies were classified as sternal-spinal-body wall complex II (SSBWC II). In the BSA Type VIII group, animals with spinal structural anomalies and no other skeletal anomaly were classified as spinal-body wall complex IV (SPBWC IV). A summary of the classified cases is given in Table 2. The phenotype observed in all BSA cases is shown in Table 3.

## 4. Discussion

Body stalk anomaly (BSA) is characterized by the presence of body wall defects, skeletal abnormalities, and an absent or abnormal umbilical cord (UC). A classification system for BSA, based on the type of body wall and skeletal anomalies, is proposed (Figure 7). All cases presented in this study were classified as BSA, except for two piglets that exhibited omphalocele as their only anomaly. All other piglets displayed a large ventral wall defect alongside spinal and UC anomalies. Building on the classification for pigs [6] and the classification for humans [2], the following classification system is proposed, identifying eight distinct types of BSA: BSA Type I: Cases with UC anomalies, anal atresia, and/or urogenital anomalies, thoracoabdominoschisis, and two structural skeletal anomalies affecting the limbs and spine (P17, P79, P240, and P278). BSA Type II: Cases with UC anomalies, anal atresia, and/or urogenital anomalies, thoracoabdominoschisis, and one structural skeletal anomaly (spinal only in this study: P168, P208, and P331). BSA Type III: Cases with UC anomalies, anal atresia, urogenital anomalies, abdominoschisis, and two structural skeletal anomalies affecting the limbs and spine (P65 and P160). BSA Type IV: Cases with UC anomalies, anal atresia, and/or urogenital anomalies, abdominoschisis, structural spinal anomalies, and no other structural skeletal anomalies (P184). BSA Type V: Cases with UC anomalies, thoracoabdominoschisis, and two structural skeletal anomalies affecting the spine and sternum (P380). BSA Type VI: Cases with UC anomalies, thoracoabdominoschisis, and either spinal (P307) or sternal structural anomalies (P226, P227, P228, P264, P267, P283, P390, P395, and P401). These last cases correspond to Cantrell syndrome, except for case P395, which did not show any cardiac defects. BSA Type VII: Cases with UC anomalies, abdominoschisis, and both sternal and spinal structural skeletal anomalies (P353). BSA Type VIII: Cases with UC anomalies, abdominoschisis, and spinal structural anomalies only (P76, P134. P285, P241, P274, and P382). P63 and P120 were classified as having omphalocele; they did not show any other anomaly.

Although many authors describe skeletal abnormalities in BSA, limb anomalies are often treated as an independent complex, referred to as the limb-body wall complex (LBWC) [10,18]. This principle is reflected in the proposed BSA classifications for both pigs [6] and humans [2], which consider the anatomical features and presumed etiology. However, no isolated LBWC cases were observed in this study; all animals with structural limb defects also had structural spinal defects and were included in the spinal-limb-body wall complex (SPLBWC). In addition to SPLBWC, three other structural skeletal complexes are proposed: Spine-body wall complex (SPBWC), sternal-body wall complex (STBWC), and sternal-spinal-body wall complex (SSBWC). For all BSA types, the structural skeletal anomalies are categorized as follows: Spinal: extreme retroflexion, extreme scoliosis, vertebral agenesis, and hemivertebrae. Thoracic: Cleft sternum, rib anomalies, and associated respiratory or cardiac anomalies. Limb: Structural anomalies such as amelia and hindlimb phocomelia. Nonstructural skeletal anomalies include spinal and thoracic deformations, limb malpositioning, amputations, and arthrogryposis.

In the case of LBWC, Russo et al. [19] identified two distinct phenotypes: the placentocranial adhesion phenotype and the placentoabdominal adhesion phenotype. Martín-Alguacil [2] considered the cranial, abdominal, and cranioabdominal phenotypes to be overlapping. In this study, of the 28 animals diagnosed with BSA, 11 animals had the abdominal phenotype, 11 had the cranial phenotype, and 7 had the cranioabdominal overlapping phenotype. In the abdominal phenotype group, all piglets showed anal atresia, except for P120, which instead presented with severe urogenital anomalies such as bilateral hydronephrosis and a hypoplastic bladder and testis, and renal ectopia and hypoplasia of the penis; it was confirmed by Martínez-Frías [20] that the presence of anal atresia is very frequent in body stalk anomalies. Furthermore, they confirmed that the association of anal atresia, spine anomalies, renal/urinary tract anomalies, and genital anomalies constitutes a group of anomalies that tended to be present together in the same child because they are pathogenically related and, since they are of blastogenetic origin, they constitute a primary polytopic developmental field [21]. All the abdominal adhesion phenotype piglets presented either renal or urinary anomalies and/or genital anomalies, confirming the association of anomalies in BSA as a primary polytopic developmental field.

Twenty-eight animals presented with the BSA features with multiple congenital anomalies, such as the curved and deformed appearance of the spine associated with UC abnormalities. UC was abnormal in all piglets, with umbilical vessels dispersed on the amniotic membrane. Three piglets showed SUA and one umbilical vein, and unilateral hypoplasia of one umbilical artery was observed in all other piglets. The presence of only two umbilical vessels was also described by Russo et al. [19] in the eight cases of placenta-abdominal adhesion phenotype. The UC is embryologically dependent on the normal closure of the ventral wall and on the embryonic folding process, which results in the failure of the umbilical ring to close and the formation of an abnormal UC [15]. Some authors mentioned that LBWC showed no gender predilection [22], but Martín-Alguacil and Avedillo [6] described seven females and only one male affected in the porcine BSA study. In the current study group, 77% (23/30) of the affected animals were females, 82% (9/11) were in the abdominal phenotype, 64% (7/11) were in the cranial phenotype, and all cases in the cranioabdominal overlapping phenotype were females.

To establish a criterion for the evaluation of structural skeletal defects, it is important to consider these anomalies embryologically. When congenital anomalies of the spine are associated with anomalies of other systems, as in the case of BSA, they are particularly of those originating in the early embryonic period [23,24]. Extreme scoliosis and/or extreme retroflexion of the spine are multifactorial anomalies that may originate in the embryonic period and are caused by defects in formation and may be associated with abnormalities in body folding, neural tube defects, and somite differentiation. Abnormalities in body folding or somite differentiation can result in extreme or misplaced curvatures of the spine. Extreme scoliosis was present in two cases, and extreme retroflexion of the spine in eight cases. Segmentation defects may be secondary. Hemivertebrae are formed during embryonic development [23] and are the result of a failure to form one sagittal half of the vertebra, including the centrum and neural arch [23,25]. The cause is unknown but is most likely related to congenital unilateral vascular deficiency [23], resulting in predominantly scoliotic angulation deformities. Incomplete formation results in a laterally wedged vertebra with varying degrees of unilateral hypoplasia; however, bilateral pedicles are present to complete the vertebral arch [23,25]. Some other anomalies, such as vertebral arch agenesis, vertebral hypoplasia, or agenesis (P79), and curled, twisted, or angled tail, observed in eight cases, are considered to be a consequence of abnormal development of the spine and are referred to as structural spinal defects. Structural sternal anomalies such as total or partial sternoschisis were observed in 10 of the cases studied. Structural sternal defects were not present in cases with thoracoabdominoschisis in BSA types I, II, III, and IV, but they were observed in one case of BSA Type V with thoracoabdominoschisis and mainly in eight cases of BSA Type VI, corresponding to the cases diagnosed with Cantrell syndrome. Rittler et al. [14], after evaluating 450 cases, concluded that AB is responsible for limb partial amputation, especially in the upper limbs, observing that phocomelia in the hind limbs predominated in cases without amniotic bands, although they only considered amelia as a structural anomaly. Arthrogryposis is characterized by multiple congenital contractures in at least two different parts of the body [26]. Congenital contractures generally occur because of a decrease in fetal movement [26,27]. The understanding of the causes of congenital malformations as complex body wall closing defects has been greatly enhanced by the rapid advances in knowledge of signaling pathways and their regulation during development [16]. Several temporally regulated mechanisms drive ventral midline closure, and the type of BWD depends on the stage at which the insult takes place. The fact that ventral midline closure relies on a dynamic TGFβ-dependent recruitment of myofibroblasts by sequential waves of cellular movements that occur during ventral body wall development may explain the diversity of phenotypes in ventral body wall closure defects [28]. In humans, anomalies of the ventral body wall are often caused by multiple factors, making it challenging to identify the exact genetic and environmental origins. Although not yet proven clinically and genetically, these anomalies may also result from somatic mutations that lead to mosaicism, where some cells in the ventral body wall carry a gene mutation [29]. Since the ventral body wall forms through extensive tissue movements involving collective cell actions, the mosaic inactivation of gene function can be particularly disruptive. Disruptions in PCP genes, which regulate large-scale tissue behaviors, can have significant effects, as seen in the early development of the thoracic wall and the closing neural tube [30,31]. BSA is not usually associated with chromosomal abnormalities; some genetic defects related to embryonic development may contribute to its occurrence [32].

In current cases of skeletal anomalies, mechanical factors and vascular disturbances that are associated with compression or entrapment of the amniotic band (AB) early in development may play a role. Disruption of the blood supply to certain regions during early development may result in abnormal growth or failure of vertebral segmentation, as seen in hemivertebrae. The presence of a midline cleft palate in P160, P307, P353, and P380 can be explained by the amniotic band action during early embryo development [33,34]. Palatoschisis can also occur because of the failure of structures to migrate or converge in the midline since the more usual clefts represent a failure of structures to merge at the midline [35]. Amniotic band syndrome (ABS) may include body wall anomalies, cranial anomalies, and limb amputation. The presence of the characteristic anomalies is enough for ABS diagnosis, even if bands are not present [15]. The presence of amniotic bands was only observed in P380. When abnormalities are associated with AB effects, it is important to determine whether they are structural or nonstructural. Late AB effects are considered nonstructural anomalies because they affect organs that are already formed, such as in limb or digit amputations. Early AB effects, on the other hand, can disrupt developmental processes and are therefore considered structural defects [23].

A rigorous classification system for BSA is essential, particularly in human medicine, to ensure accurate diagnosis, effective communication, and tailored management strategies. Despite the tendency to provide a general diagnosis for most cases, these anomalies often present with significant variations in their underlying causes, severity, and anatomical involvement [9,32,36]. In the first classification of BSA in pigs [6], four types of BSA were identified, and by using the diagnostic tool on cases from the medical literature (218 cases), the classification was extended to eight types [2]. In this work, all eight described types of BSA were diagnosed. Classifying and grading the severity of BSA can significantly enhance diagnostic and clinical practices in both veterinary and especially in human medicine in several ways. A standardized classification system helps clinicians accurately identify and diagnose BSA. This leads to earlier detection and more precise differentiation from other conditions with similar presentations. By grading the severity of cases, healthcare providers can tailor treatment plans to the specific needs of each patient. This ensures that patients receive the most appropriate level of care, whether it involves surgical intervention, supportive care, or other medical treatments. A severity grading allows for better prognostic predictions, helping clinicians and families understand the likely outcomes and potential complications. This information is crucial for making informed decisions about treatment options and long-term care. A unified classification system facilitates the collection and analysis of data across different cases and institutions. This can lead to a deeper understanding of the condition, its causes, and the effectiveness of various treatments, ultimately driving advancements in medical knowledge and practice. In veterinary medicine, similar benefits apply. An accurate classification and severity grading can improve the diagnosis and treatment of BSA in animals, leading to better health outcomes and more effective management of these conditions. A precise classification not only aids in understanding the specific developmental processes disrupted in each case but also facilitates more accurate prognostication and the development of individualized treatment plans. Moreover, it enables clinicians and researchers to identify patterns, improve diagnostic criteria, and refine therapeutic approaches, ultimately enhancing patient outcomes and advancing medical knowledge in this complex field. The proposed classification is based on anatomical features and on the presumptive etiology of the skeletal anomalies as a unifying criterion for a precise diagnosis of complex body wall anomalies when the spine, sternum, and limbs are affected, as in BSA.

## 5. Conclusions

The diagnosis of BSA encompasses a wide range of complex malformations with varying degrees of anatomical involvement. This highlights the need for a standardized classification system to ensure an accurate diagnosis, prognosis, and better understanding of these anomalies. For the first time, a classification system for BSA has been developed that incorporates both anatomical and embryological criteria, defining eight distinct types. This classification considers not only limb defects but also structural anomalies of the spine and sternum, providing a more comprehensive framework and enhancing its diagnostic utility.

The study shows that limb structural defects in BSA are consistently associated with spinal defects, forming the spinal-limb-body wall complex (SPLBWC). This finding highlights the apparent interdependence of limb and spinal anomalies in BSA cases and validates the inclusion of these combined defects in the classification.

A criterion for the classification of structural spinal defects has been established, focusing on anomalies in which the normal development of spinal elements is disrupted or altered. This criterion provides a clear reference for distinguishing between structural and nonstructural spinal anomalies, ensuring accuracy in diagnosis and classification.

## Figures and Tables

**Table 1:** Summary of the anomalies described in the studied cases and diagnosed as body stalk anomaly (BSA).

Case			BWA	UCA	SPA	LA	STA	AA	CRA/FA	UA/GA	CD	AB	Proposed Classification
**P17**	F	[6,16]	THO/Ab	DUV	ERS	Left pelvic limb phocomelia	-	+	-	Persistent urogenital sinus Hydroureters Absent urinary bladder Uterus connected to urogenital sinus	-	-	BSA I SPLBWC I
**P79**	F	[6,16]	THO/Ab	DUV	ERS Short tail	Right thoracic limb amelia	-	+	-	Persistent urogenital sinus Hydro ureters Right kidney hydronephrosis Left kidney hypoplastic Absent urinary bladder Uterus connected to urogenital sinus Hypoplastic genitalia	-	-	BSA I SPLBWC I
**P240**	F	[6,16]	THO/Ab	DUV SUA	ERS	Left thoracic limb amelia Arthrogryposis and ankyloses in pelvic limbs	-	+	-	Persistent urogenital sinus Hydro ureters Absent urinary bladder Hypoplastic genitalia	-	-	BSA I SPLBWC I
**P278**	F	[6,16]	THO/Ab	-	ERS	Left thoracic limb and pelvic limbs phocomelia	-	+	-	Hydroureters Hypoplastic urinary bladder Left kidney absent Right kidney anomalous Hypoplastic genitalia	-	-	BSA I SPLBWC I
**P168**	F	[6,16]	THO/Ab	DUV	ERS	Anomalous rotation and arthrogryposis and ankyloses in pelvic limbs	-	+	-	Unilateral right kidney agenesis	-	-	BSA II SPBWC I
**P208**	M	[6,16]	THO/Ab	DUV SUA	ERS	Arthrogryposis and ankyloses in pelvic limbs	-	+	-	Absent scrota Bilateral cryptorchidism Hypoplastic penis	-	-	BSA II SPBWC I
**P331**	F		THO/Ab	DUV	ERS	Arthrogryposis and ankyloses in pelvic limbs	-	+	-	Unilateral left kidney agenesis Hypoplastic genitalia	-	-	BSA II SPBWC I
**P65**	F	[15]	Ab	DUV SUA	S Curly tail	Ankylosis and arthrogryposis in pelvic limbs Bilateral patellar agenesia	-	+	-	Absent gall bladder Hypoplastic genitalia	-	+	BSA III SPLBWC II
**P160**	F	[6,16]	rLAB	DUV	ES Curly tail	Left thoracic limb amelia	-	+	Palatoschisis	Unilateral left kidney agenesis	-	-	BSA III SPLBWC II
**P184**	F	[6,16]	rLAB	DUV	ES	Anomalous rotation and arthrogryposis and ankyloses in pelvic limbs	-	+	-	Hypoplastic kidneys Persistent urogenital sinus Hypoplastic genitalia	-	-	BSA IV SPBWC II
**P380**	F		THO/Ab	DUV Short	Spina bifida occulta C1-C5, hypoplasia of the vertebral arches T4-T6, agenesis of the vertebral arches T7-T15	Arthrogryposis left forelimb	Sternoschisis and sternal hypoplasia (3 left sternites and 4 right sternites)	-	Cleft palate Left caudolateral cranioschisis with exencephaly Hypoplasia of the left auricle Left blepharitis. Left exophthalmia	-	-	+	BSA V SSBWC I
**P307**	F		THO/Ab	DUV	ERS	Arthrogryposis and ankyloses in pelvic limbs	-	-	Palatoschisis with bilateral cheiloschisis Hydroanacephaly Ocular malformations	-	-	-	BSA VI SPBWC III
**P226**	F	[16,17]	THO/Ab	DUV Shorter	-	-	Midline splitting except for the two first sternebrae	-	-	-	Heart globous shape with right auricle dilation, VSD	-	BSA VI STBWC Cantrell Syndrome
**P227**	M	[16,17]	THO/Ab	DUV Shorter	-	-	Midline splitting of the five more caudal sternebrae	-	-	-	ASD	-	BSA VI STBWC Cantrell Syndrome
**P228**	F	[16,17]	THO/Ab	DUV Shorter	-	-	Midline splitting of the most caudal sternebrae	-	-	-	Heart globous shape with right auricle dilation, VSD	-	BSA VI STBWC Cantrell Syndrome
**P264**	M	[16,17]	THO/Ab	DUV Shorter	-	-	-	-	-	-	Large interatrial defect, atresia of the mitral valve Single coronary artery	-	BSA VI STBWC Cantrell Syndrome
**P267**	F	[16,17]	THO/Ab	DUV Shorter	-	-	Midline splitting of the four more caudal sternebrae	-	-	-	Hypoplastic auricles, great vessel transposition, VSD	-	BSA VI STBWC Cantrell Syndrome
**P283**	F	[16,17]	THO/Ab	DUV Shorter	-	-	Midline splitting of the most caudal sternebrae	-	-	-	ASD	-	BSA VI STBWC Cantrell Syndrome
**P390**	F		THO/Ab	DUV	-	-	Sternoschisis and sternal hypoplasia	-	-	-	Cardiac ectopy Patent truncus arteriosus	-	BSA VI STBWC Cantrell Syndrome
**P395**	M		THO/Ab	DUV	-	-	Bifid (forked) sternum	-	-	-	-	-	BSA VI STBWC
**P401**	M		THO/Ab	DUV	-	-	Midline splitting of the most caudal sternebrae	-	-	-	Large interatrial defect, left atrial enlargement, right atrial hypoplasia Tricuspid valve stenosis	-	BSA VI STBWC Cantrell Syndrome
**P353**	F		Ab	DUV	KLS Angled tail	Arthrogryposis in all four limbs Hypoplastic hindlimbs. “Buddha-like” sitting position	Hypoplastic sternum	-	Cleft palate Mandibular brachygnathia	-	-	-	BSA VII SSBWC II
**P76**	F	[16]	Ab	DUV	- Short tail	Ankylosis and arthrogryposis in pelvic limbs	-	-	-	-	-	+	BSA VIII SPBWC IV
**P134**	F	[16]	Ab	DUV	- Twisted tail	Ankylosis and arthrogryposis in pelvic limbs	-	-	-	-	-	+	BSA VIII SPBWC IV
**P285**	F		Ab	DUV	MS Presence of two hemivertebrae (T15 and T16)	Arthrogryposis of the right hind limb	-	-	-	-	-	+	BSA VIII SPBWC IV
**P382**	M		rLAB	DUV Short	K Angled tail	Arthrogryposis hind limbs	-	-	-	Right unilateral cryptorchidism	-	+	BSA VIII SPBWC IV
**P241**	F	[16]	rLAb	DUV	rLS	Right forelimb phocomelia	-	-	-	-	-	-	BSA VIII SPBWC IV
**P374**	F		Ab	DUV	K Sacrococcygeal agenesis	Arthrogryposis in all four limbs	-	-	-	-	-	-	BSA VIII SPBWC IV
**P63**	F	[16]	Ab	DUV	-	-	-	-	-	-	-	-	Omphalocele
**P120**	M	[16]	Ab	-	-	-	-	-	-	Bilateral hydronephrosis and hypoplastic bladder The left testis is located in the extraembryonic coelom together with the left kidney (renal ectopia) Hypoplasia of the penis	-	-	Omphalocele

AA, anal atresia; AB, amniotic band; Ab, abdominoschisis; ASD, auricular septal defect; ASA, axial skeletal anomaly; BWA, body wall anomaly; CD, cardiac defects; CRA/FA, cranial anomaly and/or facial anomaly; DUV, dispersed umbilical vessels; ES, extreme scoliosis; ERS, extreme retroflexión of the spine; F, female; K, Kyphosis; KLS, Kypholordoescoliosis; LA, limb anomaly; M, male; MS, moderate scoliosis; OD, other defects; rLAB, right lateral abodominoschisis; rLS, right lateral scoliosis; S, scoliosis; SPA, spine anomaly; SPBWC, spinal-body wall complex; SPLBWC, spinal-limb and body wall complex; STA, sternal anomaly; SSBWC, sternal-spinal-body wall complex; STBWC, sternal-body wall complex; SUA, single umbilical artery; THO/Ab, thoracoabdominoschisis; UA/GA, urinary anomaly and/or genital anomaly UCA, umbilical cord anomaly; VSD, ventricular septal defect. The + and - signs indicate the presence or absence of anomalies.

**Table 2:** Classification by the differential features of the studied piglets.

		Anomalies	Total	Cases
BSA I/SPLBWC I		Thoracoabdominoschisis, anal atresia, genital or urinary anomalies, or structural spinal and limb anomalies.	4	P17, P79, P240, P278
BSA II/SPBWC I		Thoracoabdominoschisis, anal atresia, genital or urinary anomalies, or structural spinal anomalies.	3	P168, P208, P331
BSA III/SPLBWC II		Abdominoschisis, anal atresia, genital or urinary anomalies, or structural spinal and limb anomalies.	2	P65, P160
BSA IV/SPBWC II		Abdominoschisis, anal atresia, genital or urinary anomalies, or structural spinal anomalies.	1	P184
BSA V/SSBWC I		Thoracoabdominoschisis, no anal atresia, genital or urinary anomalies, or structural spinal and limb anomalies.	1	P380
BSA VI	SPBWC III STBWC	Thoracoabdominoschisis, no anal atresia, genital or urinary anomalies, or structural spinal anomalies. Thoracoabdominoschisis, no anal atresia, genital or urinary anomalies, or structural sternal anomalies.	10	P307 P226, P227, P228, P264, P267, P283, P390, P395, P401
BSA VII/SSBWC II		Abdominoschisis, no anal atresia, genital or urinary anomalies, or structural sternal and spinal anomalies.	1	P353
BSA VIII/SPBWC IV		Abdominoschisis, no anal atresia, genital or urinary anomalies, or structural spinal anomalies.	6	P76, P134, P241, P285, P382, P374
Omphalocele		Abdominoschisis	2	P63, P120

SPBWC, spinal-body wall complex; SPLBWC, spinal-limb and body wall complex; SSBWC, sternal-spinal-body wall complex; STBWC, sternal-body wall complex.

**Table 3:** Phenotypes of the studied cases.

Phenotype	Cases
Cranial	P226, P227, P228, P241, P264, P267, P280, P383, P390, P395, P401
Abdominal	P17, P65, P76, P134, P168, P184, P208, P285, P331, P382
Cranioabdominal overlapped	P79, P160, P240, P278, P307, P353, P374

## Data Availability

The original contributions presented in this study are included in the article. Further inquiries can be directed to the corresponding author(s).

## References

[1] Goldstein I., Winn H.N., Hobbins J.C. (1989). Prenatal diagnostic criteria for body stalk anomaly. Am. J. Perinatol..

[2] Martín-Alguacil N. (2020). Anatomy-based diagnostic criteria for complex body wall anomalies (CBWA). Mol. Genet. Genom. Med..

[3] Lockwood C.J., Scioscia A.L., Hobbins J.C. (1986). Congenital absence of the umbilical cord resulting from maldevelopment of embryonic body folding. Am. J. Obstet. Gynecol..

[4] Paul C., Zosmer N., Jurkovic D., Nicolaides K. (2001). A case of body stalk anomaly at 10 weeks of gestation. Ultrasound Obstet. Gynecol..

[5] Zeidler S., Oudesluijs G.G., Schoonderwaldt E.M., Van Bever Y. (2014). Early prenatal disruption; a foetus with features of severe limb body wall sequence, body stalk anomaly and amniotic bands. J. Genet. Couns..

[6] Martín-Alguacil N., Avedillo L. (2020). Body stalk anomalies in pig—Definition and classification. Mol. Genet. Genom. Med..

[7] Yang Y., Wang H., Wang Z., Pan X., Chen Y.J. (2020). First trimester diagnosis of body stalk anomaly complicated by ectopia cordid. Int. Med. Res..

[8] Pappalardo E., Gulino F.A., Ettore C., Cannone F., Ettore G. (2023). Body Stalk Anomaly Complicated by Ectopia Cordis: First-Trimester Diagnosis of Two Cases Using 2- and 3-Dimensional Sonography. J. Clin. Med..

[9] Ye C.H., Li S., Ling L. (2023). Analysis of characteristic features in ultrasound diagnosis of fetal limb body wall complex during 11-13^+6^ weeks. World J. Clin. Cases.

[10] Narayan R., Meena A., Sarkar R., Agrawal M. (2024). A Rare Case Report of Limb Body Wall Complex. Cureus.

[11] Laughton K.W., Fisher K.R., Halina W.G., Partlow G.D. (2005). Schistosomus reflexus syndrome: A heritable defect in ruminants. Anat. Histol. Embryol..

[12] Mateo I., Camón J. (2008). Schistosoma reflexum in a cat: Insights into aetiopathogenesis. J. Feline Med. Surg..

[13] Lunney J., Van Goor A., Walker K., Hailstock T., Franklin J., Dai C. (2021). Importance of the pig as a human biomedical model. Sci. Transl. Med..

[14] Rittler M., Campaña H., Poletta F.A., Santos M.R., Gili J.A., Pawluk M.S., Consentino V.R., Gimenez L., Lopez-Camelo J.S. (2019). Limb body wall complex: Its delineation and relationship with amniotic bands using clustering methods. Birth Defects Res..

[15] Martín-Alguacil N., Avedillo L. (2020). Body wall defects and amniotic band syndrome in pig (*Sus. scrofa domesticus*). Anat. Histol. Embryol..

[16] Martín-Alguacil N., Avedillo L. (2020). Body wall defects: Gastroschisis and omphalocoele in pigs (*Sus. scrofa domesticus*). J. Comp. Pathol..

[17] Martín-Alguacil N., Avedillo L. (2020). Cantrell Syndrome (Thoracoabdominal Ectopia Cordis; Anomalous Umbilical Cord; Diaphragmatic, Pericardial and Intracardiac Defects) in the Pig (*Sus scrofa domesticus*). J. Comp. Pathol..

[18] Adeleke O., Gill F., Krishnan R. (2022). Rare Presentation of Limb-Body Wall Complex in a Neonate: Case Report and Review of Literature. AJP Rep..

[19] Russo R., d’Armiento M., Angrisani P., Vecchione R. (1993). Limb-body wall complex: A critical review and a nosological proposal. Am. J. Med. Genet..

[20] Martínez-Frías M.L. (1997). Epidemiological characteristics of amniotic band sequence (ABS) and body wall complex (BWC): Are they two different entities?. Am. J. Med. Genet..

[21] Martínez-Frías M.L., Bermejo E., Rodríguez-Pinilla E. (2000). Anal atresia, vertebral, genital, and urinary tract anomalies: A primary polytopic developmental field defect identified through an epidemiological analysis of associations. Am. J. Med. Genet..

[22] Saritha S., Gouri G., Sumangala S. (2013). Limb body wall complex or body stalk complex or cyllosomas: A case report. Int. J. Res. Med. Sci..

[23] Tsou P.M., Yau A., Hodgson A.R. (1980). Embryogenesis and prenatal development of congenital vertebral anomalies and their classification. Clin. Orthop. Relat. Res..

[24] Westworth D.R., Sturges B.K. (2010). Congenital spinal malformations in small animals. Vet. Clin. N. Am. Small Anim. Pract..

[25] Hedequist D., Emans J. (2007). Congenital scoliosis. J. Pediatr. Orthop..

[26] Hall J.G., Vincent A. (2003). Arthrogriposis: Neuromuscular Diseases of Infancy, Childhood, Adolescence—A Clinician’s Approach.

[27] Haliloglu G., Topaloglu H. (2013). Arthrogryposis and Fetal Hypomobility Syndrome: Handbook of Clinical Neurology.

[28] Aldeiri B., Roostalu U., Albertini A., Wong J., Morabito A., Cossu G. (2017). Transgelin-expressing myofibroblasts orchestrate ventral midline closure through TGFβ signaling. Development.

[29] Formstone C., Aldeiri B., Davenport M., Francis-West P. (2024). Ventral body wall closure: Mechanistic insights from mouse models and translation to human pathology. Dev. Dyn..

[30] Mao Y., Kuta A., Crespo-Enriquez I., Whiting D., Martin T., Mulvaney J., Irvine K.D., Francis-West P. (2016). Dchs1-Fat4 regulation of polarized cell behaviours during skeletal morphogenesis. Nat. Commun..

[31] Galea G.L., Maniou E., Edwards T.J., Marshall A.R., Ampartzidis I., Greene N.D.E., Copp A.J. (2021). Cell non-autonomy amplifies disruption of neurulation by mosaic Vangl2 deletion in mice. Nat. Commun..

[32] Gică N., Apostol L.M., Huluță I., Panaitescu A.M., Vayna A.M., Peltecu G., Gana N. (2024). Body Stalk Anomaly. Diagnostics.

[33] Gupta K., Venkatesan B., Chandra T., Rajeswari K., Devi T.K. (2015). Amniotic band syndrome with sacral agenesis and umbilical cord entrapment: A case report emphasizing the value of evaluation of umbilical cord. J. Radiol. Case Rep..

[34] Muraskas J.K., McDonnell J.F., Chudik R.J., Salyer K.E., Glyn L. (2003). Amniotic band syndrome with significant orofacial clefts and disruptions and distortions of craniofacial structures. J. Pediatr. Surg..

[35] Light T.R., Ogden J.A. (1993). Congenital constriction band syndrome. Pathophysiology and treatment. Yale J. Biol. Med..

[36] Davile M., Gupta A., Parashar R., Mundle S. (2024). Deciphering Body Stalk Anomaly: A Rare Case Presentation and Review. Oman Med. J. (OMJ).

